# 2,2′-(Piperazine-1,4-di­yl)diacetonitrile

**DOI:** 10.1107/S1600536812021733

**Published:** 2012-05-19

**Authors:** Wei Gao, Jing Yang, Xin-Ling Wang, Ning Zhou, Xue-Fen Wu

**Affiliations:** a School of Pharmacy, Henan University of Traditional Chinese Medicine, Zhengzhou 450008, People’s Republic of China

## Abstract

The complete mol­ecule of the title compound, C_8_H_12_N_4_, is generated by a crystallographic inversion centre. The piperazine ring adopts a chair conformation with the N-bonded substituents in equatorial positions. In the crystal, mol­ecules are linked by C—H⋯N_c_ (c = cyanide) hydrogen bonds.

## Related literature

For related structures, see: Ma *et al.* (2007 ▶); Liu & Liu (2011 ▶); Luo & Weng (2011 ▶).
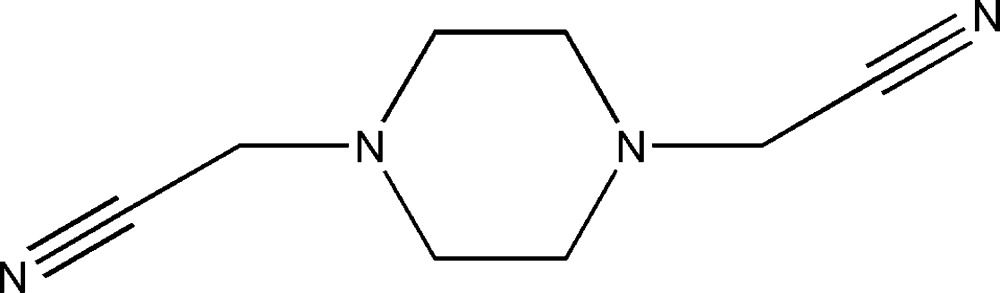


## Experimental

### 

#### Crystal data

C_8_H_12_N_4_
*M* *_r_* = 164.22
Monoclinic, 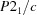

*a* = 6.3452 (13) Å
*b* = 6.6731 (13) Å
*c* = 11.077 (2) Å
β = 95.61 (3)°
*V* = 466.78 (16) Å^3^
*Z* = 2
Mo *K*α radiation
μ = 0.08 mm^−1^
*T* = 293 K
0.20 × 0.18 × 0.10 mm

#### Data collection

Rigaku Saturn CCD diffractometer
Absorption correction: multi-scan (*CrystalClear*; Rigaku/MSC, 2005 ▶) *T*_min_ = 0.985, *T*_max_ = 0.992
3739 measured reflections
1076 independent reflections
835 reflections with *I* > 2σ(*I*)
*R*_int_ = 0.032

#### Refinement

*R*[*F* ^2^ > 2σ(*F* ^2^)] = 0.046
*wR*(*F* ^2^) = 0.150
*S* = 1.05
1076 reflections
55 parameters
H-atom parameters constrained
Δρ_max_ = 0.10 e Å^−3^
Δρ_min_ = −0.15 e Å^−3^

### 

Data collection: *CrystalClear* (Rigaku/MSC, 2005 ▶); cell refinement: *CrystalClear*; data reduction: *CrystalClear*; program(s) used to solve structure: *SHELXS97* (Sheldrick, 2008 ▶); program(s) used to refine structure: *SHELXL97* (Sheldrick, 2008 ▶); molecular graphics: *SHELXTL* (Sheldrick, 2008 ▶); software used to prepare material for publication: *SHELXL97*.

## Supplementary Material

Crystal structure: contains datablock(s) global, I. DOI: 10.1107/S1600536812021733/hb6795sup1.cif

Structure factors: contains datablock(s) I. DOI: 10.1107/S1600536812021733/hb6795Isup2.hkl

Supplementary material file. DOI: 10.1107/S1600536812021733/hb6795Isup3.cml

Additional supplementary materials:  crystallographic information; 3D view; checkCIF report

## Figures and Tables

**Table 1:** Hydrogen-bond geometry (Å, °)

*D*—H⋯*A*	*D*—H	H⋯*A*	*D*⋯*A*	*D*—H⋯*A*
C3—H3*A*⋯N2^i^	0.97	2.57	3.427 (2)	147

Symmetry code: (i) 
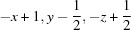
.

**Fig. 1.**
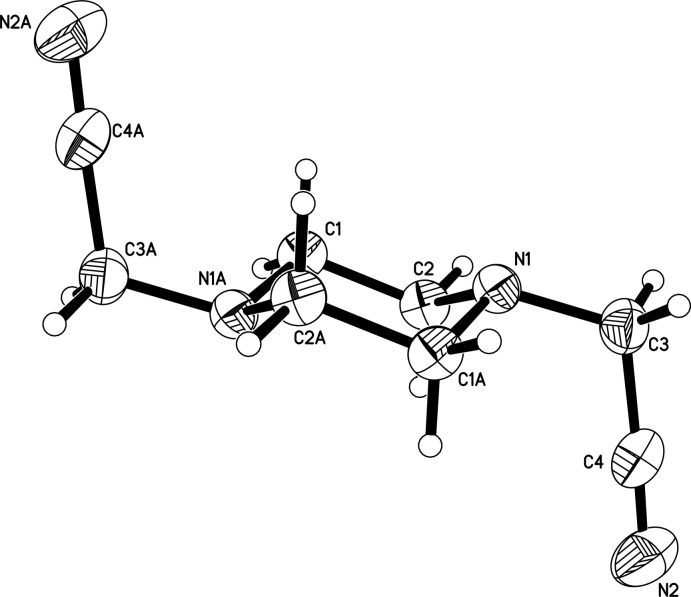
The molecular structure of (I). Displacement ellipsoids are drawn at the 30% probability level and H atoms are shown as small spheres of arbitrary radii.

**Fig. 2.**
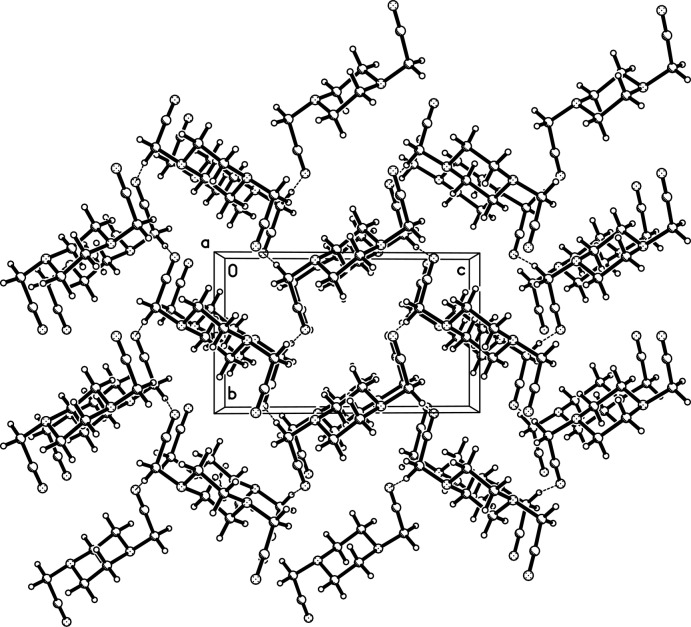
The crystal packing for (I).

## supplementary crystallographic information

### Experimental

Piperazine (25 mmol) and triethylamine (50 mmol), dissolved in 20 ml 95% of ethanol, was added dropwise to the stirred solution of chloroacetonitrile (50 mmol) at reflux. The mixture was stirred for 8 h at reflux. The mixture was stirred overnight at room temperature, evaporated in vacuum and the residue was purified by recrystallization from ethanol to give the title compound. Colourless blocks were grown from dichloromethane/ethanol solution.

### Refinement

All the H atoms were positioned geometrically (C—H = 0.93–0.97 Å) and refined as riding with *U*_iso_(H) = 1.2*U*_eq_(C) or 1.5*U*_eq_(methyl C).

### Crystal data

**Table:**

C_8_H_12_N_4_	*F*(000) = 176
*M**_r_* = 164.22	*D*_x_ = 1.168 Mg m^−^^3^
Monoclinic, *P*2_1_/*c*	Mo *K*α radiation, λ = 0.71073 Å
*a* = 6.3452 (13) Å	Cell parameters from 1156 reflections
*b* = 6.6731 (13) Å	θ = 3.1–27.8°
*c* = 11.077 (2) Å	µ = 0.08 mm^−^^1^
β = 95.61 (3)°	*T* = 293 K
*V* = 466.78 (16) Å^3^	Block, colorless
*Z* = 2	0.20 × 0.18 × 0.10 mm

### Data collection

**Table:**

Rigaku Saturn CCD diffractometer	1076 independent reflections
Radiation source: rotating anode	835 reflections with *I* > 2σ(*I*)
Confocal monochromator	*R*_int_ = 0.032
ω scans	θ_max_ = 27.9°, θ_min_ = 6.4°
Absorption correction: multi-scan (*CrystalClear*; Rigaku/MSC, 2005)	*h* = −8→5
*T*_min_ = 0.985, *T*_max_ = 0.992	*k* = −8→8
3739 measured reflections	*l* = −14→14

### Refinement

**Table:**

Refinement on *F*^2^	Primary atom site location: structure-invariant direct methods
Least-squares matrix: full	Secondary atom site location: difference Fourier map
*R*[*F*^2^ > 2σ(*F*^2^)] = 0.046	Hydrogen site location: inferred from neighbouring sites
*wR*(*F*^2^) = 0.150	H-atom parameters constrained
*S* = 1.05	*w* = 1/[σ^2^(*F*_o_^2^) + (0.0937*P*)^2^ + 0.0176*P*] where *P* = (*F*_o_^2^ + 2*F*_c_^2^)/3
1076 reflections	(Δ/σ)_max_ < 0.001
55 parameters	Δρ_max_ = 0.10 e Å^−^^3^
0 restraints	Δρ_min_ = −0.15 e Å^−^^3^

### Special details

**Table:**

Geometry. All e.s.d.'s (except the e.s.d. in the dihedral angle between two l.s. planes) are estimated using the full covariance matrix. The cell e.s.d.'s are taken into account individually in the estimation of e.s.d.'s in distances, angles and torsion angles; correlations between e.s.d.'s in cell parameters are only used when they are defined by crystal symmetry. An approximate (isotropic) treatment of cell e.s.d.'s is used for estimating e.s.d.'s involving l.s. planes.
Refinement. Refinement of *F*^2^ against ALL reflections. The weighted *R*-factor *wR* and goodness of fit *S* are based on *F*^2^, conventional *R*-factors *R* are based on *F*, with *F* set to zero for negative *F*^2^. The threshold expression of *F*^2^ > σ(*F*^2^) is used only for calculating *R*-factors(gt) *etc*. and is not relevant to the choice of reflections for refinement. *R*-factors based on *F*^2^ are statistically about twice as large as those based on *F*, and *R*- factors based on ALL data will be even larger.

### Fractional atomic coordinates and isotropic or equivalent isotropic displacement parameters (Å^2^)

**Table:**

	*x*	*y*	*z*	*U*_iso_*/*U*_eq_	
N1	0.06045 (14)	0.54542 (14)	0.12377 (8)	0.0464 (4)	
N2	0.3536 (2)	0.9796 (2)	0.16532 (15)	0.0904 (6)	
C1	−0.21559 (17)	0.52728 (19)	−0.04400 (11)	0.0497 (4)	
H1A	−0.2823	0.4143	−0.0078	0.060*	
H1B	−0.3249	0.6036	−0.0910	0.060*	
C2	−0.10710 (17)	0.65762 (18)	0.05439 (11)	0.0499 (4)	
H2A	−0.0474	0.7746	0.0184	0.060*	
H2B	−0.2095	0.7030	0.1079	0.060*	
C3	0.1569 (2)	0.6568 (2)	0.22683 (12)	0.0584 (4)	
H3A	0.2562	0.5701	0.2741	0.070*	
H3B	0.0476	0.6945	0.2779	0.070*	
C4	0.2700 (2)	0.8402 (2)	0.19376 (13)	0.0647 (4)	

### Atomic displacement parameters (Å^2^)

**Table:**

	*U*^11^	*U*^22^	*U*^33^	*U*^12^	*U*^13^	*U*^23^
N1	0.0471 (6)	0.0463 (6)	0.0461 (5)	0.0023 (4)	0.0062 (4)	−0.0010 (4)
N2	0.0816 (10)	0.0743 (10)	0.1129 (13)	−0.0215 (7)	−0.0029 (9)	−0.0028 (8)
C1	0.0410 (6)	0.0516 (7)	0.0570 (7)	0.0054 (4)	0.0062 (5)	−0.0018 (5)
C2	0.0465 (6)	0.0479 (7)	0.0561 (7)	0.0087 (4)	0.0092 (5)	−0.0032 (5)
C3	0.0657 (8)	0.0575 (8)	0.0512 (7)	−0.0009 (6)	0.0013 (5)	−0.0049 (5)
C4	0.0592 (8)	0.0618 (9)	0.0707 (9)	−0.0035 (6)	−0.0057 (6)	−0.0112 (7)

### Geometric parameters (Å, º)

**Table:**

N1—C3	1.4471 (16)	C1—H1B	0.9700
N1—C2	1.4567 (15)	C2—H2A	0.9700
N1—C1^i^	1.4680 (14)	C2—H2B	0.9700
N2—C4	1.1305 (19)	C3—C4	1.4824 (19)
C1—N1^i^	1.4681 (14)	C3—H3A	0.9700
C1—C2	1.5071 (17)	C3—H3B	0.9700
C1—H1A	0.9700		
			
C3—N1—C2	112.51 (10)	C1—C2—H2A	109.6
C3—N1—C1^i^	112.82 (9)	N1—C2—H2B	109.6
C2—N1—C1^i^	110.49 (9)	C1—C2—H2B	109.6
N1^i^—C1—C2	109.89 (9)	H2A—C2—H2B	108.2
N1^i^—C1—H1A	109.7	N1—C3—C4	114.00 (10)
C2—C1—H1A	109.7	N1—C3—H3A	108.8
N1^i^—C1—H1B	109.7	C4—C3—H3A	108.8
C2—C1—H1B	109.7	N1—C3—H3B	108.8
H1A—C1—H1B	108.2	C4—C3—H3B	108.8
N1—C2—C1	110.06 (9)	H3A—C3—H3B	107.6
N1—C2—H2A	109.6	N2—C4—C3	178.07 (16)
			
C3—N1—C2—C1	−174.46 (8)	C2—N1—C3—C4	−64.42 (14)
C1^i^—N1—C2—C1	58.45 (13)	C1^i^—N1—C3—C4	61.41 (14)
N1^i^—C1—C2—N1	−58.10 (13)	N1—C3—C4—N2	13 (5)

Symmetry code: (i) −*x*, −*y*+1, −*z*.

### Hydrogen-bond geometry (Å, º)

**Table:**

*D*—H···*A*	*D*—H	H···*A*	*D*···*A*	*D*—H···*A*
C3—H3*A*···N2^ii^	0.97	2.57	3.427 (2)	147

Symmetry code: (ii) −*x*+1, *y*−1/2, −*z*+1/2.
